# Retraction: Long non-coding RNA H19 induces hippocampal neuronal apoptosis *via* Wnt signaling in a streptozotocin-induced rat model of diabetes mellitus

**DOI:** 10.18632/oncotarget.28576

**Published:** 2024-06-03

**Authors:** Yu-Hao Zhao, Tie-Feng Ji, Qi Luo, Jin-Lu Yu

**Affiliations:** ^1^Department of Neurosurgery, The First Hospital of Jilin University, Changchun, P.R. China; ^2^Department of Radiology, The First Hospital of Jilin University, Changchun, P.R. China

**This article has been retracted:** Upon conducting a follow-up study on the effects of five different lncRNA H19 transcripts (h19-202, h19-209, h19-204, h19-203 and h19-210) on inducing apoptosis of hippocampal neurons in diabetic rats and Wnt signaling pathway involvement, the authors did not receive consistent results and cannot support previously reached conclusions. Therefore, with the agreement of all authors, the Scientific Integrity office at Oncotarget has decided to retract this paper.

Original article: Oncotarget. 2017; 8:64827–64839. 64827-64839. https://doi.org/10.18632/oncotarget.17472

